# Robotic Approach to Ureteral Endometriosis: Surgical Features and Perioperative Outcomes

**DOI:** 10.3389/fsurg.2018.00051

**Published:** 2018-09-04

**Authors:** Andrea Giannini, Silvia Pisaneschi, Elisa Malacarne, Vito Cela, Franca Melfi, Alessandra Perutelli, Tommaso Simoncini

**Affiliations:** ^1^Division of Obstetrics and Gynecology, Department of Experimental and Clinical Medicine, University of Pisa, Pisa, Italy; ^2^Multidisciplinary Center of Robotic Surgery, Azienda Ospedaliero Universitaria Pisana, Pisa, Italy

**Keywords:** robotic, ureteral endometriosis, da Vinci Xi, da Vinci Si, gynecologic surgery, minimally invasive surgery

## Abstract

**Introduction:** Surgical treatment of ureteral endometriosis is necessary to relieve urinary symptoms of obstruction and to preserve renal function. Which surgical approach to ureteral endometriosis should be considered the most appropriate is debated, due to the lack of scientific evidence. The aim of the present study is to assess the feasibility and to describe the perioperative outcomes of minimally invasive treatment of deep ureteral endometriosis using robotic assistance, highlighting the technical benefits and the limits of this approach.

**Method:** A case-series including 31 consecutive patients affected by high-stage endometriosis including ureteral endometriosis using robotic assistance in our Department between November 2011 and September 2017.

**Results:** All procedures were successfully completed by robotic technique, resulting in full excision of the parametrial nodules involving the ureter. Mean operating time was 184.8 ± 81 min. Mean hospital stay was 4.02 ± 3 days. Perioperative complications occurred in five patients and 4 out of 5 involved the urinary tract.

**Conclusions:** Robotic surgery for deep infiltrating endometriosis of the ureter was feasible and allowed complete resection of ureteral nodules in all cases. No intraoperative complications arose, but a non-negligible rate of urinary tract complications was detected. This calls for a careful assessment of the benefits and specific risks associated with the use of robotic surgery for the treatment of deep infiltrating endometriosis of the ureter.

## Introduction

Deep-infiltrating endometriosis (DIE) affects about 20% of patients with endometriosis. Its clinical behavior is extremely heterogeneous ranging from an asymptomatic finding to a severe disorder involving multiple extragenital organs and causing symptoms as infertility, deep dyspareunia or chronic pelvic pain in 50% of cases (1). DIE negatively impacts the physical status of patients, their psychological and social quality of life (2). The urinary tract is involved in approximately 0.3–12% of patients with endometriosis, thus representing one of the most common extra-genital system affected. The bladder is the most common location involved in about 80% of cases while ureteral endometriosis is present in 14% of cases. Ureteral endometriosis is frequently classified as extrinsic or intrinsic according to histologic results and it is usually unilateral. Extrinsic ureteral endometriosis is the most usual finding, occurring in 80% of cases. It is characterized by the infiltration of the peritoneum, the uterosacral ligament, the ureteral adventitia and the surrounding connective tissue, determining extrinsic compression of the ureteral wall due to fibrosis and the related peri-ureteral desmoplastic reaction. Intrinsic ureteral endometriosis is less common and involves the mucosa or muscolaris propria (3–8).

Clinical presentation of ureteral lesions is various, and it relates to the location and size of the endometriotic lesion. A correct diagnosis is crucial because frequently few or even no symptoms can attend this disease with possible silent complications such as different degrees of hydronephrosis up to the loss of renal function. Hence, patients may experience non-specific abdominal pain, with 50% of patients lacking urinary symptoms at the time of clinical assessment. To identify ureteral endometriosis a broad variety of imaging techniques, such as magnetic resonance, ultrasound, computed tomography scan and intravenous retrograde pyelography have been suggested. However, diagnosis of ureteral endometriosis is still challenging and often a definitive assessment can be achieved only laparoscopically and histologically (9–11).

Surgery is most often necessary to treat ureteral endometriosis, with a variety of treatment options based on the size and location of the lesion and with the general aim to isolate and free the ureter from the endometriotic nodules. This is needed in order to address urinary obstruction and to preserve renal function (3). In the last 20 years, a broad body of literature confirms that ureterolysis can be adequately achieved by laparoscopic approach with acceptable complications rates (ranging from 0 to 31%) and low risk of recurrences (ranging from 0 to 12%). However, these results largely depend on the operator's dexterity and experience together with the size, depth, and extension of ureteral endometriosis. Consequently, which mini-invasive approach provides the best technical aid to the surgeon still represents a matter of debate (3, 12–14). This is particularly timely given the broad diffusion of robotic-assisted mini-invasive surgery in gynecology. Indeed, it could be predicted that any platform that provides a better visualization and increases manual dexterity may come handy in the surgical management of a difficult surgery such as that for ureteral endometriosis. However, no such evidence exists in the literature.

The purpose of the present report was to describe the feasibility, safety, technical implications and perioperative outcomes of excisional surgery for ureteral endometriosis using robotic-assisted mini-invasive surgery with different da Vinci platforms.

## Materials and methods

### Patients selection and clinical assessment

The present study is a retrospective review of 31 consecutive patients who underwent robotic-assisted laparoscopic excision of endometriosis with ureteral involvement from November 2011 to September 2017. Patients were identified using ICD-9 selecting code of robotic-assisted endometriosis. All patients with intraoperative visualization and histological confirmation of Deep Infiltrating Endometriosis (DIE), who underwent robotic-assisted laparoscopic endometriosis with ureteral involvement using the da Vinci robotic platforms were included in the study. Exclusion criteria were treatment by traditional laparoscopy or abdominal route. Patients demographics such as age, parity, body mass index (BMI) and American Society of Anesthesiologists score (ASA) were derived from detailed electronic chart review. Electronic patient's files yielded information on fertility, past surgical history, previous hormonal treatments, preoperative examinations, and presence of symptoms at admission. Operating time, amount of blood loss, intraoperative complications and other relevant info related to the surgical procedures were recorded. Post-operative items such as length of hospitalization, recovery of urinary and bowel function were noted. Related perioperative (within 7 days after surgery), early post-operative (from 7 to 28 days after surgery), and late post-operative (>28 days after surgery) complications were evaluated according to the Clavien-Dindo classification criteria (15).

All patients were enrolled in the endometriosis center of our hospital, where a comprehensive clinical history is collected during clinical assessment of the disease. The charts include data concerning patient symptoms, including dysmenorrhea, dyspareunia, dyschezia, dysuria, constipation or diarrhea, chronic or peri-menstrual rectal or urological bleeding and infertility.

The severity of symptoms was evaluated on a 10-point visual analog scale (VAS), with a value of 10 as the worst pain suffered and one corresponding to minimal pain perceived.

Current and past hormonal or non-hormonal therapies were noted. In patients taking hormonal therapy in preparation for surgery such as estroprogestin oral, vaginal or patch compounds, progestins only or GnRH analogs the type and duration of therapy was documented.

An accurate vaginal clinical examination was performed in order to evaluate deep pelvic tissues involvement. If DIE was strongly suspected, a rectal examination is performed for the assessment of the disease and the planning of further instrumental investigations. During the collection of an exhaustive history of endometriosis and related symptoms, we paid particular attention to urinary symptoms. Bilateral Giordano maneuvers were carried out to induce or worsen pain in latent renal dilatation. All patients underwent preoperative transvaginal and transabdominal ultrasound assessment by skilled operators to preoperatively assess the disease and its related anatomical disruption.

When ureteral involvement was suspected, a full evaluation of the urinary tract was performed, in the search of signs of ureteral dilatation or hydronephrosis. In all such cases the urinary system was also assessed with multislice computed tomography scans or, alternatively, abdominal magnetic resonance imaging both with intravenous injection of contrast medium and late-phase acquisitions to investigate the whole urinary tract, to rule out renal or ureteral dilatation. Hydronephrosis was defined as any degree of the renal calyx or pelvis dilatation. In order to evaluate the degree of urterohydronephrosis, intravenous pyelography was frequently performed and kidney scintigraphy was also performed to assess renal function in presence of cortical atrophy. In some cases of patients with ureterohydronephrosis or in patients with assumed complex surgery or risks factors the surgeon chose to proceed to preoperative ureteral stenting. Cystoscopy was also performed in the case of suspected bladder localization (9, 13, 14, 16–21). Detection of serum level of creatinine before and after surgery was done in all patients.

All patients were preoperatively admitted in the Department of Obstetrics and Gynecology of S. Chiara University Hospital of Pisa for endometriosis assessment and preoperative clinic evaluations. Surgery was performed in the operating rooms of the Multidisciplinary Center of Robotic Surgery of Cisanello University Hospital of Pisa using da Vinci Si and Xi robotic systems (Intuitive Surgical Inc., Sunnyvale, CA, USA). All the patients received surgery by gynecologic surgeons trained in advanced mini-invasive techniques with high expertise and skills in robotic approach. Severity and stage of endometriosis were determined during surgery using the revised American Fertility Society (rAFS) score system (22). The choice to use the robotic platform to perform the procedure was left to the surgeons, based on their experience. All patients enrolled for surgery had an extensive preoperative counseling about the risks and benefits of surgery for deep pelvic endometriosis signed a written informed consent.

All patients were examined 3 months and 6 months post-operatively, and yearly thereafter by the same group of physicians. The institutional review board of the University of Pisa approval was obtained for this study.

### Surgical preparation and technique

All patients received mechanical bowel preparation and a single dose of prophylactic antibiotic (Cefuroxime 1 g) 1 h before skin incision; antithrombotic prophylaxis was administered, if required, with low-molecular-weight heparin 12 h before surgery. A Foley catheter was placed before beginning of surgery and removed the day after. Surgery was performed using both da Vinci Si and Xi surgical systems. After general anesthesia was given, the patient was placed in a modified dorsolithotomic position, with a 26° Trendelenburg position during the procedure. The Veress needle was inserted at the umbilical level to insufflate the abdomen. After pneumoperitoneum was obtained, a umbilical 12 or 8 mm (depending on robotic platform) incision was made and the trocar was placed through the umbilical incision and two other 8-mm lateral ports were placed under direct visualization. A 10-mm assistant port was also inserted. Pneumoperitoneum was kept at 8–12 mmHg. The robotic surgical system was docked using three arms and single-docking full robotic technique: camera port through the umbilical incision and two robotic instrument ports through the lateral incisions. A uterine manipulator was positioned to manipulate the uterus from below. Robotic tools used during the procedure included monopolar scissors, Maryland or fenestrated bipolar forceps, a large needle holder, and a mega suture cut needle holder if sutures were required. After induction of pneumoperitoneum and insertion of the 30° robotic camera, the whole abdominal cavity was explored. First, adhesions limiting access to the pelvis were removed. Isolation of the involved ureter was performed in all patients with DIE in order to approach safely every pelvic localization of the disease, such as lateral and/or posterior parametrium nodules. Lysis of adhesions allowed surgeons to achieve full access to the pelvis, to free the ovaries and to fix them to the abdominal wall with simple sutures or using dedicated devices to gain proper visualization of the surgical field. Nerve visualization began with the identification of the ureters at their cross with the common iliac artery. In case of endometriosis around the ureter, the opening of the retroperitoneum, starting at a distance of 1–2 cm medially from this cross allowed obtaining a good exposure of the space. The pararectal space was developed by peeling apart the leaf of the broad ligament, making a small opening in the characteristic areolar tissue at the basis of the broad ligament, and performing a gentle dissection with robotic bipolar forceps and monopolar scissors into the pararectal space so that the Latzko and Okabayashi spaces were laid open.

In detail, when the ureter was extrinsically involved by an endometriotic lesion of the parametrium, in order to achieve surgical radicality, the medial and the lateral pararectal space, (Okabayashi's and Latzko's pararectal space) were developed from the peritoneum of the sacral promontory to the lateral parametrium predominantly using robotic bipolar forceps as a dissector. Starting from the pelvic brim, ureterolysis was performed along the course of the ureter on the pelvic sidewall until healthy tissue was reached. This surgical task allowed ideal identification of the hypogastric and uterine vessels, isolation of the hypogastric nerves (HN) from the pelvic brim to the pelvic plexus, detachment of the ureter, and identification of the deep uterine vein that represents an anatomic-surgical landmark useful for the identification of the plane which divides, ventrally and cranially, the parametrial vascular portion from the neural part, dorsally and caudally.

When lateral parametrium was involved, the ureter was isolated up to its cross over the uterine artery, thus obtaining a parametrial tunnel. The uterine artery was spared during these operative steps; this was often feasible, with the exception of those cases with a broad involvement of the cardinal ligament. After this surgical step, medial visualization of the lateral part of the superior hypogastric plexus (SHP) and the HN followed. Identification of the HN at the origin allowed a conservative approach during radical excision of endometriosis of the visceral afferent and efferent nerves to the bladder, uterus, vagina, and dorsally to the rectum. In order to preserve these fibers, they were separated from the uterosacral ligaments, which were then cut when necessary. All endometriotic nodules, including lesions involving the uterosacral ligaments, peritoneum, the torus of the uterus and vagina were excised.

**Figure 1:**
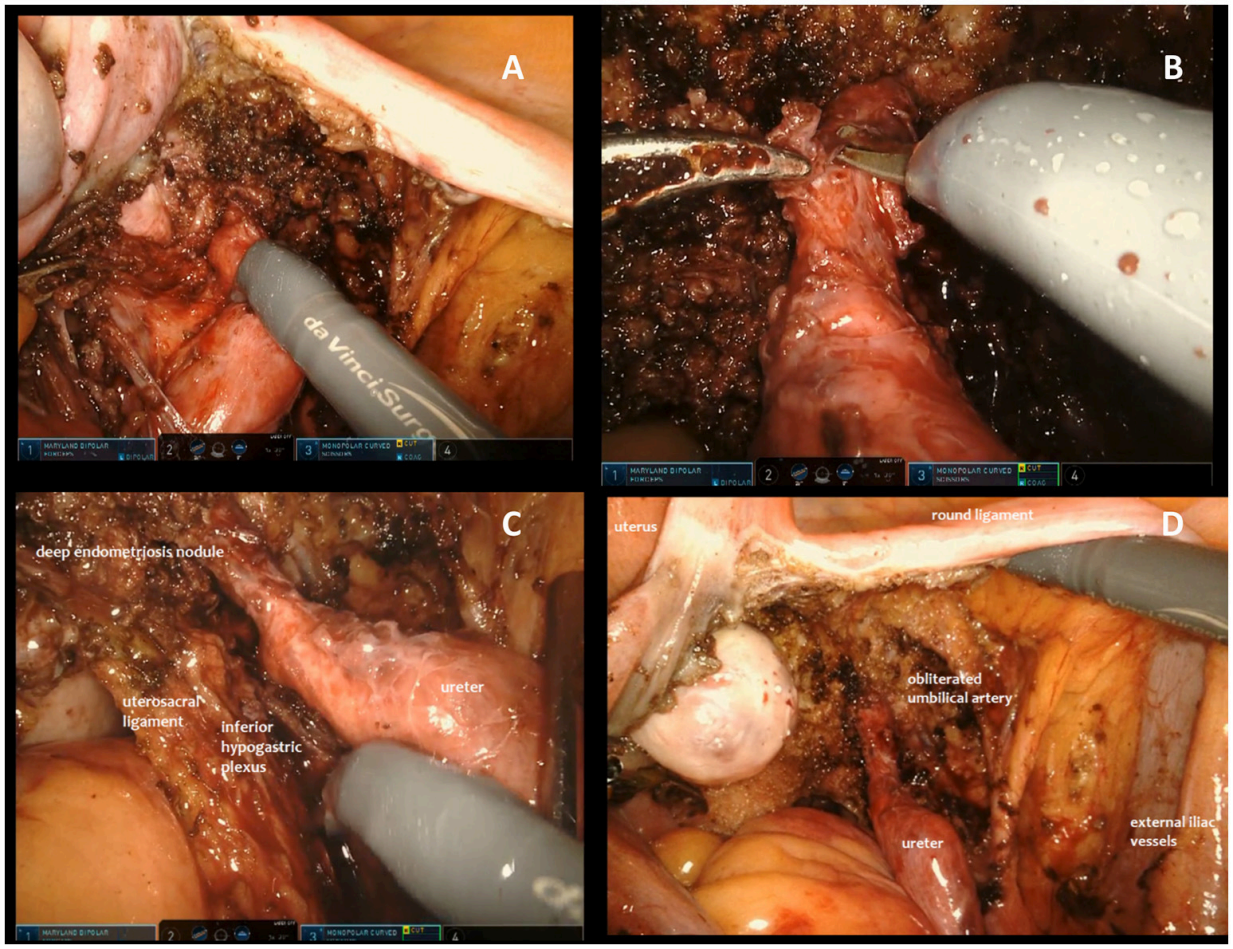
Different steps of surgical excision of endometriotic nodule involving the ureter. **(A)** Opening of the broad ligament and identification of the right ureter involved by an extrinsic lesion. **(B)** In case of extrinsic ureteral endometriosis, ureterolysis is accomplished using bipolar forceps and monopolar scissors until anatomical structures seem completely free from disease. **(C)** During the procedure, care is taken to minimize the risk of ureteral resection and injuries to surrounding structures, with a particular attention to preserving the nerves. **(D)** When skeletonization is concluded an accurate check of the whole pelvic ureter is performed to identify intraoperative lesions.

In conclusion, in the case of extrinsic ureteral endometriosis ureterolysis could be accomplished using bipolar forceps and monopolar scissors until all anatomic structures were completely free from disease. During all procedures, care was taken to minimize the risk of ureteral resection. When skeletonization was concluded an accurate check of the whole pelvic ureter was performed to identify intraoperative lesions. If a ureteral lesion was detected or strongly suspected in a patient who didn't undergo preoperative ureteral stenting, a stent was placed intraoperatively by cystoscopy at the end of robotic treatment and then removed after 60 days in order to decrease the risk of fistula. In the case of an intraoperative ureteral injury the surgeon extemporarily decided whether to perform a robotic suture or to perform a ureteral resection with re-anastomosis. This surgical choice depended also on whether the endometriotic nodule involved intrinsically the mucosa or muscolaris. In this series of robotic procedures, all additional pelvic endometriotic lesions were removed during the same surgery (Figure 1A–D).

### Statistical analysis

Sample characteristics were determined using descriptive statistics. Continuous variables were expressed as the mean, median and range, whereas categorical variables were expressed as counts and percentages. The statistical package SPSS®, version 17 (Windows, SPSS Inc., Chicago, Il USA) were used for data analysis.

## Results

From November 2011 to September 2017, 31 patients with high-stage pelvic endometriosis including ureteral involvement underwent robotic-assisted surgery. Surgeries were performed by three different surgeons (TS, AP, VC) with advanced skills in robotic surgery. The patients had a mean age of 39.1 ± 4.5 years and mean Body Mass Index (BMI) of 23 ± 2.6 Kg/m^2^. Twelve patients (21.7%) had undergone previous surgery for endometriosis. Twenty-one patients (67.7%) complained of dysmenorrhea, 20 (64.5%) of dyspareunia, 13 (41.9%) of urinary tract symptoms (dysuria and hydronephrosis, hydroureteronephrosis) and 8 (12.9%) of bowel symptoms. Ureteral stenting before surgery was performed in 9 patients (29%). At the time of surgery 20 patients (64.5%) took hormonal therapy (estroprogestins, progestin-only pills, and GnRH analogs). The characteristics of the patients are reported in Table 1.

**Table 1:** Patients characteristics.

	***n* (%)**
N. of case	31
Age (years ± SD)	39.1 ± 4.56
BMI (kg/m2 ± SD)	22.97 (21.75%)
History of endometriosis surgery	12 (38.7%)
**PARITY**	
Nulliparus	20 (64.5%)
Primiparus or multiparus	11 (35.4%)
**SYMPTOMS**	
Dysmenorrhea	21 (67.7%)
Dyspareunia	20 (64.5%)
Urinary tract signs	13 (41.9%)
Digestive signs	8 (25.8%)
**ASA SCORE**	
I	2 (6.4%)
II	25 (80.6%)
III	4 (12.9%)
IV	0
Perioperative hormonal treatment	20 (64.5%)
Preoperative stenting	9 (29%)

*ASA, American Society of Anesthesiologists*.

Mean operating time was 184.8 ± 81 min (range 70–330 min) and estimated blood loss 207 ± 142 ml (range 35–430 ml). None of the patients required an intraoperative blood transfusion.

In 28 cases (90.3%) surgeons preferred three robotic arms whereas in 3 (9.7%) cases the fourth arm was used. Surgeons chose side docking in 29 cases (93.5%) and central docking in 2 cases (6.5%). Right side docking was preferred in 22 cases of 29 (75.8%) whereas left side docking in 7 cases (24.2%). Robotic ureterolysis was associated in 6 cases (19.3%) to a recto-vaginal nodule resection, in 24 cases (77.4%) to resection of the uterosacral ligament and in 7 cases (22.6%) to excision of ovarian endometriomas. In 21 cases the endometriotic nodule involved the left ureter while in 10 cases the right ureter was involved.

Ureterolysis was successfully completed in all cases. In all cases, the resection of the endometriotic nodule surrounding or involving the ureter was considered to be complete, with no residual endometriotic tissue.

Five patients (16%) reported immediate intraoperative or post-operative complications; four out of five involved the urinary tract. Ureteral injury occurred in one patient, requiring robotic ureterovesical reimplantation after intraoperative stenting (during the same surgery). During surgery for concomitant ureteral and uterosacral endometriosis one ureteral fistula occurred after a ureteral injury during shaving of the nodule from the ureteral wall. The patients had not received preoperative ureteral stenting. In this case a second operation was performed to repair the fistula. One patient developed a paravaginal hematoma treated conservatively with antibiotic prophylaxis. No complications related to the duration and the steepness of the Trendelenburg position neither to robotic trocars or instruments occurred during all procedures. Total reoperation rate was 6.4% (*n* = 2). The average length of stay was 4 days. Histopathology reports confirmed the presence of endometriotic tissue in all cases (Table 2).

**Table 2:** Perioperative results.

	**Patients *n* 31 (%)**
Operating time (min)	184.8 ± 81
Estimated blood loss	207 ± 142
Hospital stay (days)	4.02 ± 3
Full robotic technique	31 (100%)
**ROBOTIC ARMS**	
Three arms	28 (90.3%)
Four arms	3 (9.7%)
Side Docking	29 (93.5%)
Right side	22 (75.8%)
Left side	7 (24.2%)
Central docking	2 (6.5%)
**ASSOCIATED SURGERY**	
Rectovaginal nodules	6 (19.3%)
Uterosacral ligaments	24 (77.4%)
Endometriomas	7 (22.6%)
**URETERAL ENDOMETRIOSIS**	
Left	21 (67.7%)
Right	10 (32.3%)
**MAIN COMPLICATIONS**	
Ureteral fistula	2 (6.4%)
Hydronephrosis	1 (3.2%)
Vaginal hematoma	1 (3.2%)
Ureterovescical reimplantation	1 (3.2%)
Histopathology confirmation	31 (100%)

## Discussion

Our experience using the robotic approach to ureteral endometriosis shows that the procedure is feasible, extremely reproducible and safe with rates of complications that are in line with the literature. All procedures were completely performed by robotic technique, notwithstanding the complexity of the surgical procedures, involving patients with high rates of previous surgery and multicompartmental, high-stage endometriosis. In our series, 38.7% of patients who underwent robotic treatment had a previous history of endometriosis surgery and 41.9% of patients claimed urinary tract symptoms before surgery. Moreover, a consistent proportion of our population (64.5%) was receiving perioperative hormonal treatment at the time of surgery with no significant improvement of symptoms. Indeed, in our series, robotic ureterolysis was associated in 6 cases (19.3%) to recto-vaginal nodule resection, in 24 cases (77.4%) to resection of the uterosacral ligament and in 7 cases (22.6%) to excision of ovarian endometriomas. This highlights the fact that our population is representative of the typical clinical scenario of a chronic or recurrent disease often with previous surgical procedures that is the common presentation of the patient with DIE of the ureter.

It well-appreciated how ureteral endometriosis (and more in general DIE) is challenging from a surgical standpoint. An extensive knowledge of anatomy and of its variability, as well as appropriate surgical skills are mandatory to approach this disease. This is even more relevant due to the fact that the improvement of symptoms is closely related to the radicality of the surgical excision of the endometriotic nodules. Thus, any tool that may facilitate safe and effective dissection of DIE is possibly important to improve the success of the procedures.

The da Vinci robotic surgical platform (Intuitive Surgical Inc., Sunnyvale, CA, USA) may be a rational step forward to achieve a safe and radical minimally invasive surgery for ureteral endometriosis. The robotic platform has recently changed laparoscopy, adding increased magnification with its 10X view/three-dimensional vision, 7° of freedom of the instruments, and physiologic tremor filtering. These peculiarities provide surgeons with an improved ergonomic setting, simplifying complex laparoscopic steps in the narrow operative field of the pelvis such as suturing or performing dissection, thus facilitating anatomical identification and preservation of critical structures without compromising the radicality of surgery (23–25).

Relevant to this point of view, in all our cases a complete excision of the parametrial nodule involving the ureter was achieved, with no residual disease left. This may highlight how the robotic platform may be useful to achieve those complex dissections and surgical maneuvers that are requested to obtain a complete isolation of the ureter and a satisfactory excision of the parametrial nodules.

However, increased radicality often carries the inherent risk of an increased complication rate, which is not desirable in the face of the young age of the typical patient and of the aim of surgery, which is always to improve quality of life. Indeed, the distorted anatomy of the pelvis with endometriosis and the technical limits of traditional laparoscopy still make radical pelvic surgery extremely challenging with rates of urogenital complications described for conventional laparoscopy that compare to those of open surgery (19, 25–27). In 2009 Bosev et al. published the largest series on laparoscopic approach to ureteral endometriosis observing 1% of ureteral complications. Later series demonstrated that in expert hands laparoscopic ureterolysis can be successfully accomplished with a slight rate of perioperative complications (10, 12, 14, 16, 21). Uccella et al. in 2014 showed even lower rates (<1%) of intraoperative ureteral injuries and long-term complications in a high-risk patients series, thus confirming that with an adequate level of surgical expertise, ureteral endometriosis can be effectively managed laparoscopically (19).

At the same time, evidence reporting on whether the robotic approach to ureteral DIE may be safer is weak and controversial (27–29). Only one study published by Nezhat et al. compared perioperative outcomes of conventional and robotic laparoscopic treatment of endometriosis whatever the stage of disease in a series of 78 patients. However, only 5% of the patients had stage 4 endometriosis. The authors described no conversion to laparotomy and similar outcomes for the two different minimally invasive approaches with longer operating times in the robotic group. The authors did not demonstrate any advantage of robotic resection of early stages of endometriosis thus recommending such surgery for stage 3 and 4 (25). Interestingly, a recent international multicentric retrospective study by Collinet evaluated perioperative outcomes of 164 women with stage 4 endometriosis who underwent robotic treatment. They described one conversion to laparotomy, two bowel injuries and two ureteral fistulae after ureterolysis with a reoperation rate of 1.8%. In addition, this is the only report on robotic surgery for ureteral endometriosis evaluating reproductive outcomes. After surgical treatment, 41.2% of women had a desire for pregnancy, and 28.2% of them became pregnant (28). In 2011, the group of Brudie described the outcomes of a series of 80 patients who underwent robotic surgery for stage 4 endometriosis but only in 36.3% of surgeries ureterolysis was performed. This series reported shorter operating time, similar re-operation rate but a significantly higher rate of conversion to laparotomy (5%) compared with the multicentric report of Collinet (29).

In our experience, two patients (6.4%) presented post-operative ureteral fistula needing re-intervention; one (3.2%) developed hydronephrosis and one patient (3.2%) underwent ureterovesical re-implantation thus demonstrating that the rate of complications is not negligible with this technique and should be further explored.

In discussing why a surgical platform that allows better view, enhanced dexterity and increased precision may still be associated with urinary tract complications a set of items should be taken in mind. From a technical standpoint, many surgeons dislike the absence of tactile feedback during the initial use of the robotic console, however, magnification of the images thanks to the three-dimensional vision fills in this gap and increases the precision of accurate dissection around delicate anatomical structures such as the ureter, the rectum, the bladder or nerves without compromising the identification and the radical dissection of endometriotic nodules (28). This peculiar way of compensating for tactile absence may be relevant to the development of complications. The main surgical difficulty in these procedures is the dissection of severe adhesions around sensitive structures such as the ureter, the bladder, the rectum or nerves. It is plausible to hypothesize that the two cases of ureteral fistula and the lesion of ureter that required re-implantation may have been facilitated by the lack of tactile feedback, possibly allowing the surgeon to exert excessive strength during ureteral shaving. In our series, preoperative stenting was performed only in 9 (29%) patients. If lack of tactile accuracy represents a pitfall, a possible counter-measure could be represented by pre-surgical ureteral stenting in all women enrolled for robotic treatment.

A second technical issue with robotic surgery for ureteral DIE may possibly be represented by the extensive use of energy to achieve dissection that is typical of robotic surgery. To date, there is an absolute lack of literature concerning the degree of lateral heat spread with robotic monopolar, bipolar and ultrasonic tools. Only a paper by Lukas J. et coll. demonstrates that coagulation instruments used in robotic laparoscopic surgery have different thermal spreads depending on power setting and application time, however, the authors did not test the instruments in gynecologic surgery (30).

The main weakness of our study, as with all the previously published series, relates to the number of patients. In fact, we have to acknowledge that the retrospective design and the modality of the collection of outcomes do not allow obtaining information on long-term and reproductive outcomes. Furthermore, this report does not describe quality of life after surgery.

Our study shows that robotic assistance allows complete, radical, excision of ureteral endometriosis in complex cases. Robotic surgery is safe in terms of intraoperative outcomes but the rate of urinary tract complications is not negligible. Careful attention should be dedicated in the coming years to understand correctly the surgical differences of robotic-assisted as compared to standard laparoscopy. This is particularly relevant in terms of safety and complication rates that may be modified specifically by the robotic platform, which has an impact on surgical manipulations and mode of energy use.

In conclusion, whether robotic assistance may improve the outcomes of patient with ureteral DIE remains to be explored. Nevertheless, our results show that robotic surgery is highly effective in terms of radicality of excision of endometriotic nodules involving the ureters, with non-negligible rates of post-operative urinary tract complications.

## Ethics statement

The protocol was approved by the Ethical Commette of the University of Pisa. All subjects gave written informed consent in accordance with the Declaration of Helsinki.

## Author contributions

All authors (AG, SP, EM, VC, FM, AP, and TS) gave substantial contributions to the conception or design of the work; or the acquisition, analysis, or interpretation of data for the work. They contributed during drafting the work or revising it critically for relevant intellectual content and for final approval of the version to be published. They had an agreement to be accountable for all aspect of the work in ensuring that questions related to the accuracy or integrity of any part of the work are appropriately investigated and resolved.

### Conflict of interest statement

The authors declare that the research was conducted in the absence of any commercial or financial relationships that could be construed as a potential conflict of interest.
